# Reducing Outpatient Waiting Time: A Simulation Modeling Approach

**DOI:** 10.5812/ircmj.7908

**Published:** 2013-09-05

**Authors:** Afsoon Aeenparast, Seyed Jamaleddin Tabibi, Kamran Shahanaghi, Mir Bahador Aryanejhad

**Affiliations:** 1 Department of Health Services Management, Mother and Child Health Research Center, Iranian Institute for Health Sciences Research, ACECR, Tehran, IR Iran; 2 Clinical Research Center, Milad Hospital, Tehran, IR Iran; 3 School of Management Science and Medical Information, Tehran University of Medical Sciences and Health Services, Tehran, IR Iran; 4 College of Industrial Engineering, Iran University of Science and Technology, Tehran, IR Iran

**Keywords:** Outpatients, Patients, Simulation

## Abstract

**Objectives:**

The objective of this study was to provide a model for reducing outpatient waiting time by using simulation.

**Materials and Methods:**

A simulation model was constructed by using the data of arrival time, service time and flow of 357 patients referred to orthopedic clinic of a general teaching hospital in Tehran. The simulation model was validated before constructing different scenarios.

**Results:**

In this study 10 scenarios were presented for reducing outpatient waiting time. Patients waiting time was divided into three levels regarding their physicians. These waiting times for all scenarios were computed by simulation model. According to the final scores the 9th scenario was selected as the best way for reducing outpatient's waiting time.

**Conclusions:**

Using the simulation as a decision making tool helps us to decide how we can reduce outpatient's waiting time. Comparison of outputs of this scenario and the based- case scenario in simulation model shows that combining physician's work time changing with patient's admission time changing (scenario 9) would reduce patient waiting time about 73.09%. Due to dynamic and complex nature of healthcare systems, the application of simulation for the planning, modeling and analysis of these systems has lagged behind traditional manufacturing practices. Rapid growth in health care system expenditures, technology and competition has increased the complexity of health care systems. Simulation is a useful tool for decision making in complex and probable systems.

## 1. Introduction

Over the past thirty years the dramatic increase in the cost of healthcare has compelled researchers and healthcare professionals to examine ways to improve operational efficiency and reduce costs. Discrete-event simulation is one tool available to health care decision makers which can assist in this endeavor. Discrete-event simulation is a technique which allows end users (i.e. hospital administrator, clinic manager) to assess the efficiency of existing health care delivery system, to ask: "what if" questions, and to design new systems. Discrete – event simulation can be also used to forecast the impact of changes in patient flow, to examine resource needs, and to investigate the complex relationships among the different model variables. This information allows managers to select management alternatives that can be used to reconfigure existing systems, improve system performance, or design and plan new system, without altering the present system (1). In recent years the application of simulation in healthcare has become increasingly more wide spread. Several studies reported the organizational benefits and cost savings of applying simulation to hospital planning and scheduling from a macro perspective (2-8). Other applied simulation efforts have focused attention on the operational process flow of health care delivery and capacity modeling (1, 2, 6-21). Most of the models reported are of discrete parts of hospitals, such as emergency rooms, clinics and operating theatres (22).

The review of literatures showed that the simulation of complex, integrated and multifacility systems were performed rarely. The major reasons for this gap were at first the level of complexity in these models, and secondly the resource requirements including the time and money needed to conduct such researches. So most reported studies are unit specific and facility specific. These case studies are examples of what can be performed or in some cases of what might be performed. Although the results of these case studies could not be generalized to other different systems exactly, by the way the results proposed insights for others if the papers provide general and conceptual descriptions of their approach with enough detail to permit others to use their approaches, if not their models (22). Most studies working on outpatient services focus on reducing or managing patient waiting time, because waiting and treatment time are usually regarded as important determinants of patient satisfaction and service quality. Reducing outpatient's waiting time is not only valuable for patients but also helpful to decrease the hospital workload (22-24).

The simulation models are actually used for real decision making in health care. So these studies are used not only for estimating patient's waiting time but also for predicting the impact of different solutions in reducing waiting time before implementing them in real system. These studies used 3 main fields for reducing waiting time: appointment scheduling, facilitating patient flow and altering human resources or service capacity (8, 10, 11, 13-15, 17, 18, 20-24). This paper provides a simulation model for reducing outpatients waiting time in a general teaching hospital in Tehran, Iran. The specific unit of analysis was the orthopedic outpatient clinic of this hospital. Ambulatory and nonurgent patients are referred to this clinic. Patients were grouped into two main groups including new patients and follow up patients. Follow up patients were divided into two subgroups: entirely outpatient and previously inpatient. New patients and follow up outpatients must be scheduled and previously inpatients can be visited without appointment. The first group of patients (New patients and follow up outpatients) were visited by experienced residents and the second group of patients (previously inpatient follow up) at first visited by novice residents. Resident physicians may consult with their senior staff physician for diagnosis and treatment of some patients, who then joined them and discussed medical issues related to the patients. 

## 2. Material and Methods

The study was conducted in 2 phases. The first phase was a descriptive study; in this study all parameters including flow patterns in the system and mean service time in different stations were calculated. The study population was outpatients referred to hospital clinic. Convenience sampling method was used for selecting study samples. For eliminating bias in the study, patients who used different ways from usual patients such as hospital employees and their relevant were excluded from the study. At data collection stage the arrival time, service time and flow of 375 patients from arrival to exit were recorded in designed checklists. After data collection, statistical analyses including Run test descriptive statistics and some analytical test were performed by using SPSS.

The second phase was model construction study. In this study a simulation model was designed to predict changes in patients waiting time and physicians' idle time due to changes in system. The discrete process- based simulation model was created using AweSim. AweSim is a general-purpose simulation system which takes advantage of the latest windows technology to integrate programs and provide component ware. AweSim includes the Visual SLAM simulation language to build network, sub network, discrete event and continuous models ( 25 ). To validate the system aspects of the model simulation, result after running 1000 replication, was compared with actual data of system. The comparison showed that there was no statistical difference between simulation results and actual system. The validation results are presented in Table 1. 

**Table 1.** Comparison of Simulation Result With Actual Data

Service Delivery Station	Average Waiting Time (Actual Data)	S.D. of Waiting Time (Actual Data)	Average Waiting Time (Simulation Result)	P Value
**Registration**	1.5	1.96	1.462	0.672
**Cash**	0.7	1.02	0.705	0.749
**Novice resident examination**	60.67	49.89	59.602	0.561
**Experienced resident examination**	54.89	44.27	54.027	0.566
**Senior staff physician**	56.91	46.68	49.522	0.654
**Para clinic services**	42.69	21.66	41.192	0.279

After model validation, different scenarios were presented to assess the impact of different changes in outpatients waiting time. Scenarios were developed according to system situation, article review and system experts' opinions. Implemented scenarios were:

Scenario1- Increasing the number of novice residents from 2 to 3

Scenario 2- Increasing the number of experienced residents from 1 to 2 

Scenario 3- Changing the time of resident physician's attendance at the clinic from 10 to 9 A.M. and duration of their attendance at the clinic from 200 minute to 260 minute 

Scenario 4- Changing the time of senior staff physician's attendance at the clinic from 10:45 to 10 A.M and duration of their attendance at the clinic from 100 minute to 160 minute 

Scenario 5- Changing the start of patient's admission from 7:30 to 8 A.M 

Scenario 6- Mix of scenarios 1 and 2 

Scenario 7- Mix of scenarios 3 and 4 

Scenario 8- Mix of scenarios 1 and 2 and 5 

Scenario 9- Mix of scenarios 3 and 4 and 5 

Scenario 10- Mix of scenarios 1 and 2 and 3 and 4

### 2.1. Analysis

The finding of the first phase indicated that distribution of patient's entrance to the system was consistent with exponential distributions but the interval of patients arrival had a normal distribution (mean= 4.2 min, S.D. = 5.8 min). The mean service time for patients was estimated 1.13 min (S.D. = 0.44) in admission and 0.20 min (S.D. = 0.3) in cash. Patients consulted with novice residents, experienced resident and senior staff physicians (attends) according to their situation. The mean service times for these physicians were 3.46 minutes (S.D. = 2.06), 4.91 minutes (S.D. = 0.93), and 4.20 (S.D. = 0.93) minutes respectively. The outputs of base case scenario (present situation) showed that patients wait 1.55 minutes for admission, 0.68 minutes for payment, 59.60 minutes for visiting by novice resident, 54.03 minutes for visiting by experienced resident, and 49.52 minutes for visiting by attend. 

**Figure 1.**
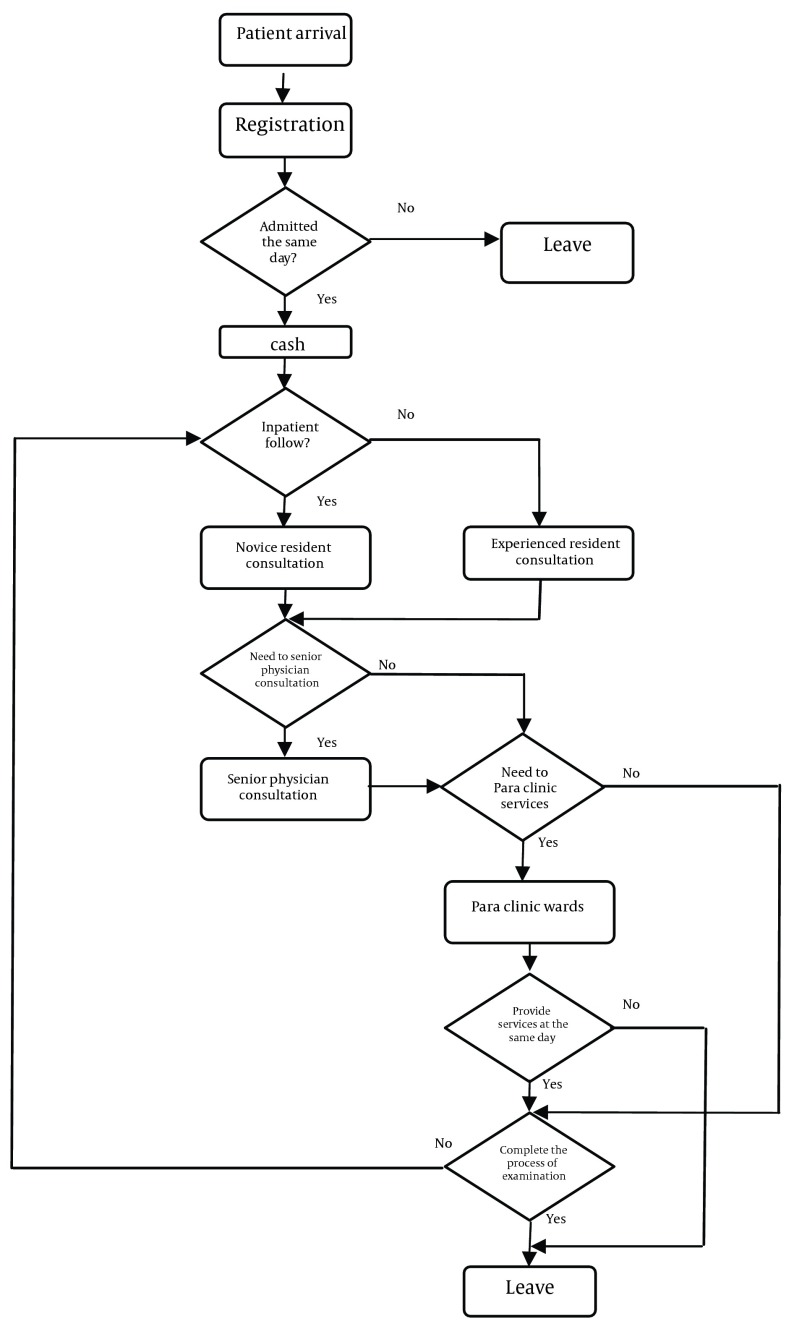
Flow Chart of Outpatient Visit in Subject Clinic

For reducing patients waiting time, different scenarios were presented. These scenarios provide some changes in physician's number, the start time of examination room, and start time of patient's admission to decrease outpatient waiting time. Some mixed scenarios also presented by considering the result of basic scenarios. After running 1000 replication of each scenario, patient's waiting time for each scenario was analyzed. These results are presented in Table 2. We consider patients waiting time in 3 levels (novice resident, experienced resident and senior staff physician). To some up 3 results different weights were used for different servers with respect to their percent of patient's (weight 2 for novice and experienced resident and weight 1 for senior staff physician), and then used weighted mean for comparison of scenarios. Analyzing the result of simulation shows that all scenarios reduce outpatients waiting time. These results are shown in Table 3. Despite the 4th scenarios would reduce senior staff total waiting time more than others, the 9th scenarios would reduce all waiting times more than others. This scenario provides some changes on residents and attends entrance time at the clinic and a small change in admission start time. This scenario would decrease present waiting time (55.36 minute) about 71.40 percent (Figure 1). 

**Table 2.** Patients Waiting Time to Minute in Different Scenarios

	Base case	Scenario 1	Scenario 2	Scenario 3	Scenario 4	Scenario 5	Scenario 6	Scenario 7	Scenario 8	Scenario 9	Scenario 10
**For examination by novice resident before attendance of resident at the clinic**	32.42	32.46	32.41	12.15	32.40	22.27	32.35	12.17	22.42	12.04	12.10
**For examination by novice resident after attendance of resident at the clinic**	27.18	18.48	9.22	6.97	27.02	15.66	8.68	7.06	5.06	1.43	1.96
**Total waiting time for examination by novice resident**	59.60	50.94	41.63	19.12	59.42	37.93	41.03	19.23	27.48	13.47	14.06
**For examination by experienced resident before attendance of resident at the clinic**	32.42	32.46	32.41	12.15	32.40	22.27	32.35	12.17	22.42	12.04	12.10
**For examination by experienced resident after attendance of resident at the clinic**	21.61	5.21	18.45	9.37	22.41	15.25	5.075	10.93	3.77	2.46	2.49
**Total waiting time for examination by experienced resident**	54.03	37.67	50.86	21.52	54.81	37.52	37.43	23.10	26.19	14.50	14.59
**For examination by senior staff Physician before physician attendance at the clinic**	24.39	28.32	30.30	51.80	13.09	25.75	32.78	15.52	32.36	19.68	17.61
**For examination by senior staff Physician after physician attendance at the clinic**	25.13	33.74	33.87	33.30	9.76	27.70	34.24	14.93	30.80	3.53	17.50
**Total waiting time for examination by senior staff physician**	49.52	62.06	64.17	85.03	22.87	53.45	67.02	30.45	63.16	23.21	35.11

**Table 3.** Weighted Mean of Waiting Times for 3 Servers

	Base case	Scenario 1	Scenario 2	Scenario 3	Scenario 4	Scenario 5	Scenario 6	Scenario 7	Scenario 8	Scenario 9	Scenario 10
**Total waiting time for examination by novice resident**	59.60	50.94	41.63	19.12	59.42	37.93	41.03	19.23	27.48	13.47	14.06
**Total waiting time for examination by experienced resident**	54.03	37.67	50.86	21.52	54.81	37.52	37.43	23.10	26.19	14.50	14.59
**Total waiting time for examination by senior staff physician**	49.52	62.06	46.17	85.03	22.87	53.45	67.02	30.45	63.16	23.21	35.11
**Weighted mean**	55.36	47.86	49.83	33.26	50.26	40.87	44.79	23.02	34.10	15.83	18.48

## 3. Conclusions

Computer simulation is an efficient approach to study complex systems. This article has shown how to use it in the study of outpatient setting management to decrease patient's waiting time. Several studies indicate that one important aspects of providing a simulation model is demonstrating the performance of healthcare facility by accurately capturing current system processes and providing a basis for improving processes of the system (1, 8). In this study we tried to go beyond this matter and use a simulation model for reducing outpatient's waiting time. Several studies in outpatient settings provide a simulation model for this reason. Some of them only focused on appointment systems (10, 14, 20), and others focused on number and schedule of providers especially physicians (10, 21). This study has tried to combine these aspects.

One important fact which is shown in table 2 is that over a half of patients waiting time is before physician's entrance at the clinic. It means that patients come to clinic soon or physicians come to clinic late. With reducing this gap patients waiting time would be reduced considerably. As is clear in some scenarios we reduced this gap to less than 10 minutes. Sum up simulation results by using weighted mean provide a clear criteria for comparing all scenarios. Analyzing the results of simulation shows that changing in physicians work time more than increasing the number of physicians has reduced patient's waiting time (comparison scenario 6 and scenario 7). It seems that combining physician's work time changing with patient's admission time changing (scenario 9) would decrease waiting time by %71.40 and is the best scenario among others for reducing outpatient waiting time.

In general this study showed that some minor changes in service setting would release patients from long waiting time. It is proposed that presenting these objective results to service providers and physicians is a useful way to improve physician attitude toward their important role in timing of clinic work and the impact of their delay on patient's waiting time. On the other hand considering physicians' punctuality as one of their evaluation factors would motivate them to be more on time. These could be important steps for reducing physician's delay and improving outpatient setting management.
